# Attitude and behavior toward bystander cardiopulmonary resuscitation during COVID-19 outbreak

**DOI:** 10.1371/journal.pone.0252841

**Published:** 2021-06-23

**Authors:** Kah-Meng Chong, Jiun-Wei Chen, Wan-Ching Lien, Mei-Fen Yang, Hui-Chih Wang, Sot Shih-Hung Liu, Yen-Pin Chen, Chien-Yu Chi, Marvin Chih-Hsien Wu, Cheng-Yi Wu, Edward Che-Wei Liao, Edward Pei-Chuan Huang, Hsien-Chin He, Hsiang-Wen Yang, Chien-Hua Huang, Patrick Chow-In Ko

**Affiliations:** 1 Department of Emergency Medicine, National Taiwan University Hospital, National Taiwan University College of Medicine, Taipei, Taiwan; 2 Department of Emergency Medicine, National Taiwan University Hospital Hsin-Chu Branch, Hsinchu, Taiwan; 3 Department of Emergency Medicine, National Taiwan University Hospital Yunlin Branch, Yunlin County, Taiwan; 4 Department of Family Medicine, National Taiwan University Hospital, National Taiwan University College of Medicine, Taipei, Taiwan; 5 Graduate Computer-Aided Engineering Group, Civil Engineering, National Taiwan University, Taipei, Taiwan; 6 Institute of Epidemiology and Preventive Medicine, National Taiwan University, Taipei, Taiwan; Fondazione IRCCS Policlinico San Matteo, ITALY

## Abstract

**Background:**

Outbreaks of emerging infectious diseases, such as COVID-19, have negative impacts on bystander cardiopulmonary resuscitation (BCPR) for fear of transmission while breaking social distancing rules. The latest guidelines recommend hands-only cardiopulmonary resuscitation (CPR) and facemask use. However, public willingness in this setup remains unknown.

**Methods:**

A cross-sectional, unrestricted volunteer Internet survey was conducted to assess individuals’ attitudes and behaviors toward performing BCPR, pre-existing CPR training, occupational identity, age group, and gender. The raking method for weights and a regression analysis for the predictors of willingness were performed.

**Results:**

Among 1,347 eligible respondents, 822 (61%) had negative attitudes toward performing BCPR. Healthcare providers (HCPs) and those with pre-existing CPR training had fewer negative attitudes (p < 0.001); HCPs and those with pre-existing CPR training and unchanged attitude showed more positive behaviors toward BCPR (p < 0.001). Further, 9.7% of the respondents would absolutely refuse to perform BCPR. In contrast, 16.9% would perform BCPR directly despite the outbreak. Approximately 9.9% would perform it if they were instructed, 23.5%, if they wore facemasks, and 40.1%, if they were to perform hands-only CPR. Interestingly, among the 822 respondents with negative attitudes, over 85% still tended to perform BCPR in the abovementioned situations. The weighted analysis showed similar results. The adjusted predictors for lower negative attitudes toward BCPR were younger age, being a man, and being an HCP; those for more positive behaviors were younger age and being an HCP.

**Conclusions:**

Outbreaks of emerging infectious diseases, such as COVID-19, have negative impacts on attitudes and behaviors toward BCPR. Younger individuals, men, HCPs, and those with pre-existing CPR training tended to show fewer negative attitudes and behaviors. Meanwhile, most individuals with negative attitudes still expressed positive behaviors under safer measures such as facemask protection, hands-only CPR, and available dispatch instructions.

## Introduction

Timely bystander cardiopulmonary resuscitation (BCPR) is vital to improve the survival of patients experiencing out-of-hospital cardiac arrest [1, 2]. Although many strategies have been developed [3, 4], BCPR is currently insufficient in most emergency medical service (EMS) systems [5, 6]. The epidemic outbreak of an emerging infectious disease (EID), such as COVID-19, may have a more negative impact on the BCPR rate for patients experiencing out-of-hospital cardiac arrest [7–10] because of the fear of getting infected while breaking social distancing rules [11, 12] and helping such patients by coming in close proximity. Although the actual risk of severe acute respiratory syndrome coronavirus 2 transmission to a health rescuer during BCPR is debatable [13], the American Heart Association and European Resuscitation Council have recently provided an interim guideline for basic life support in this situation. The use of a facemask or cloth covering the nose and mouth of a lay rescuer and patient is recommended [14, 15]. Before appropriate strategies are designed and applied to optimize the BCPR rate, we need to determine the public’s perceptions and reactions to this issue.

To the best of our knowledge, there are limited studies evaluating the impact of EIDs on bystander willingness to perform cardiopulmonary resuscitation (CPR) [16–19]. According to Ajzen’s theory of planned behavior [20], the willingness to perform CPR (i.e., behavioral intention and behavior itself) is shaped by an individual’s attitude, perceived social pressure, and perception of their capability to perform the behavior. During the COVID-19 outbreak, BCPR is considered a risky behavior in the prevailing social atmosphere [12]. This study aimed to evaluate the attitude and behavior of the public toward BCPR during an EID outbreak.

## Materials and methods

### Study setting

This study was conducted in Taiwan after the World Health Organization (WHO) declared COVID-19 as a public health emergency of international concern on January 30, 2020. Taiwan is a developed island country with a 23.59 million population spread across a total land area of 36,197 km^2^ (a population density of approximately 651 inhabitants per square kilometer), which was expected to have the second highest number of COVID-19 cases owing to its proximity to and number of flights to and from China [21]; however, it has had a low incidence of confirmed COVID-19 cases [22].

The Taiwan Central Epidemic Command Center has followed the WHO Outbreak Communication Guidelines for operations on trust and transparency, early announcement, planning, and involving and informing the public to intensify its risk communication systems [23, 24]. An official press conference with a live webcast has been held by the Central Epidemic Command Center at least once a day to update the public on the latest developments of confirmed COVID-19 cases, accumulated deaths, and critical illness. In addition, the broadcasted information included personal preventive measures, social distancing principles, updated regulations, penalties, and legitimacy concerning quarantine or illicit rumors during the COVID-19 outbreak [21, 25]. Traditional media outlets, such as newspapers, televisions, posters, and radios, as well as new social media platforms, such as Facebook, Line, Instagram, and YouTube, have been extensively used for public education and announcements [24, 26]. Owing to intense media coverage, the social atmosphere during the epidemic has changed markedly.

### Study design

We conducted a cross-sectional study via an Internet survey that aimed to evaluate the attitude and behavior of the general public toward performing BCPR during an EID outbreak. We developed a structured questionnaire that was reviewed and modified under the consensus reached by three emergency physicians who are also qualified as EMS medical directors with over 5-year EMS experiences, two experienced paramedics (with over 5-year EMS practice), two CPR-trained nonmedical laypersons, and one survey expert and statistician to improve clarity and brevity.

Our study was conducted using an unrestricted volunteer Internet survey using a self-administered method [27]. The Internet survey questionnaire (S1 Appendix) consisted of four main parts: Part A consisted of three mandatory questions regarding pre-existing training, attitude, and behavior toward performing BCPR; Part B consisted of two mandatory questions regarding the respondents’ occupational identity and residential region; Part C consisted of two mandatory questions regarding informed consent for participation in this study and for provision of personal information; and Part D consisted of five optional questions regarding the respondents’ personal information, including age, gender, and contact information (name, email, and phone number). To achieve a better response rate, we employed the following rule: If the respondents wished to participate in this Internet survey study but not disclose their personal information, then Part D could be skipped.

For data collection and better dissemination of the survey, the Internet questionnaire was created using Google Forms and linked with widely used social media platforms, including Facebook, Line, and the Bulletin Board System [28]. The questionnaire was distributed in open online forums, all of which were COVID-19- or first aid-related discussion groups. To avoid repeated submission, the respondents were required to login to access the questionnaire and were limited to providing one response only. The questionnaire responses were considered valid only if all mandatory questions were answered completely. The exclusion criteria applied were as follows: (a) unobtained informed consent, (b) nonpermanent residency in Taiwan, and (c) indefinable free-form text data in the answers.

The National Taiwan University Hospital’s Institutional Review Board approved the waiver of written informed consent in this study (Institutional Review Board number: 202008019RINA). The details of the online informed consent are shown in S1 Appendix.

### Data analysis

Statistical analyses were performed using the Statistical Package for the Social Sciences (version 24). Pearson’s chi-square test was used for categorical variable analysis. A multivariate logistic regression analysis was performed to analyze the predictors for the attitude, behavior, and willingness toward BCPR and their odds ratio (OR) with 95% confidence interval (CI). The process measures included age, gender, pre-existing CPR training, and occupational identity. Weighting was performed to adjust imbalances in the study sample, so that the weighted data could be more representative of the general population in the study region. The raking method was used for weighting based on the updated 2020 census data of the general population in Taiwan in terms of gender and age.

## Results

**Fig 1:**
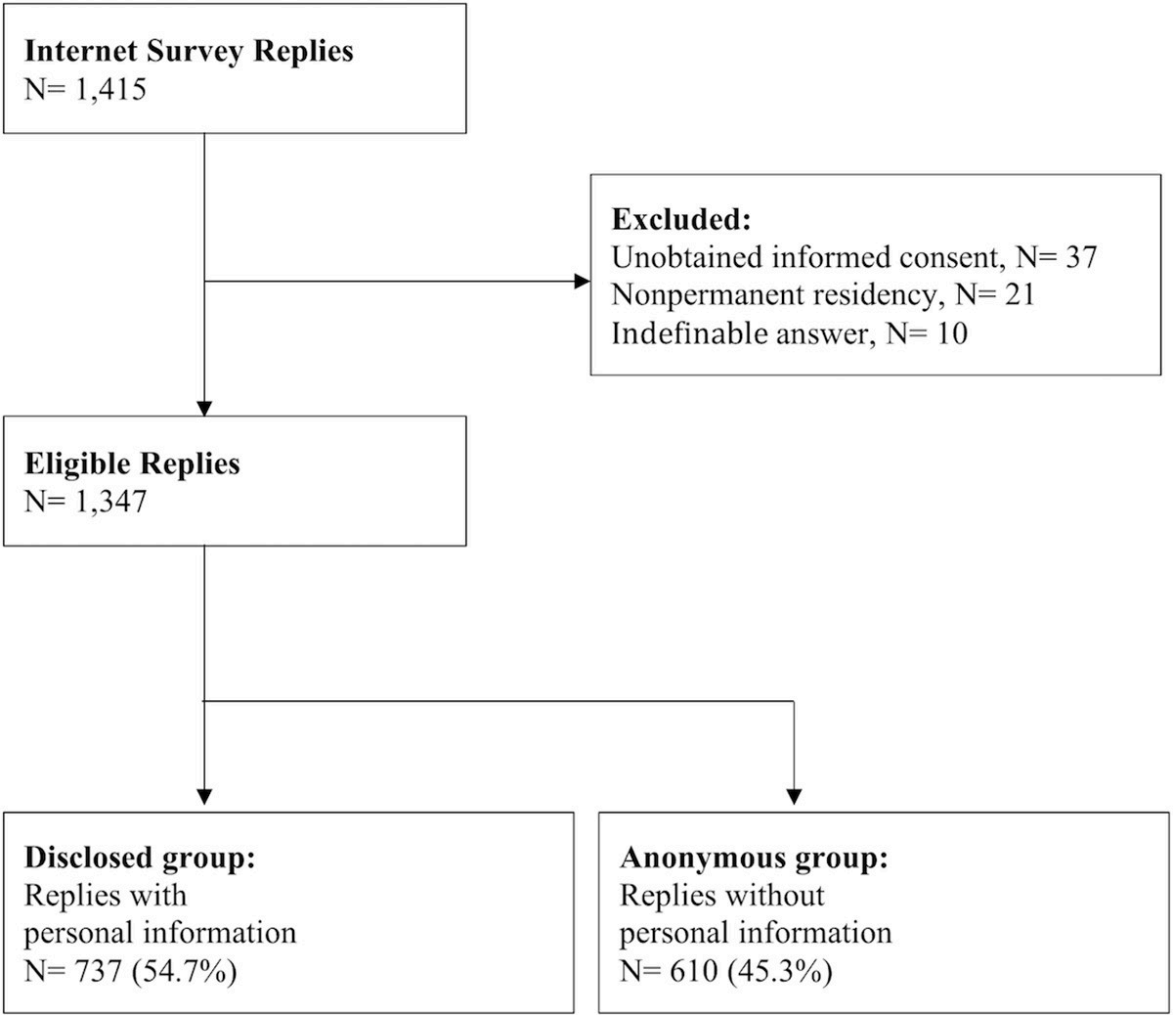
Flowchart of data collection.

A total of 1,415 replies were obtained from the Internet survey. Sixty-eight respondents (4.8%) were excluded owing to informed refusal, nonpermanent residency, and invalid answers. A total of 1,347 respondents were eligible for the data analysis (Fig 1).

### Characteristics of the respondents

Among the 1,347 eligible respondents, 807 (59.9%) were laypersons; 450 (33.4%), healthcare providers (HCPs); and 90 (6.7%), physicians. A total of 960 (71.3%) respondents reported that they had attended conventional CPR training courses, whereas 211 (15.6%) respondents had learned CPR from nonconventional educational platforms, such as online media (Table 1).

**Table 1:** Characteristics of survey respondents: All eligible, unweighted disclose, and weighted disclose groups.

		All eligible	Disclosed group (unweighted)	Disclosed group (weighted)
		n (%)	n (%)	n (%)
**Total**		1347 (100%)	737 (100%)	737 (100%)
**Gender**				
	Female	315 (23.4%)	316 (42.9%)	371 (50.3%)
	Male	421 (31.3%)	421 (57.1%)	366 (49.7%)
	Not responded	611 (45.4%)	n/a	n/a
**Age**				
	10–19	33 (2.4%)	33 (4.5%)	80 (10.9%)
	20–29	159 (11.8%)	159 (21.6%)	111 (15.1%)
	30–39	174 (12.9%)	174 (23.6%)	126 (17.1%)
	40–49	177 (13.1%)	177 (24.0%)	133 (18.0%)
	50–59	172 (12.8%)	172 (23.3%)	129 (17.5%)
	60–69	18 (1.3%)	18 (2.4%)	108 (14.7%)
	70–79	4 (0.3%)	4 (0.5%)	50 (6.8%)
	Not responded	610 (45.3%)	n/a	n/a
**Occupation**				
	Layperson	807 (59.9%)	432 (58.6%)	479 (65.0%)
	Healthcare provider	450 (33.4%)	241 (32.7%)	209 (28.4%)
	Physician	90 (6.7%)	64 (8.7%)	49 (6.6%)
**Attitude toward BCPR**				
	Not changed	525 (39.0%)	304 (41.2%)	279 (37.9%)
	Negative changed	822 (61.0%)	433 (58.8%)	458 (62.1%)
**Behavior(s) toward BCPR**				
	Will not CPR anyway	130 (9.7%)	55 (7.5%)	67 (9.1%)
	Will CPR with instruction	133 (9.9%)	78 (10.6%)	112 (5.2%)
	Will CPR with face mask	317 (23.5%)	174 (23.6%)	159 (21.6%)
	Will CPR without MTM	540 (40.1%)	283 (38.4%)	257 (34.9%)
	Will CPR anyway	227 (16.9%)	147 (19.9%)	142 (19.3%)
**Preexisting CPR training**				
	No	176 (13.1%)	107 (14.5%)	127 (17.2%)
	Conventional course	960 (71.3%)	520 (70.6%)	487 (66.1%)
	Nonconventional platform	211 (15.7%)	110 (14.9%)	123 (16.7%)

BCPR: bystander cardiopulmonary resuscitation; MTM: mouth-to-mouth breathing.

There were 610 (45.3%) respondents who preferred full anonymity and did not disclose personal information, including gender and age. There were fewer women (42.9%) and a higher proportion from the 20–29- (21.6%), 30–39- (23.6%), 40–49- (24%), and 50–59-year (23.3%) age groups in the disclosed subgroup including 737 (54.7%) respondents than in the general population included in the 2020 census. The disclosed subgroup was weighted by age and gender from the general population in the 2020 census and is presented in Table 1 (disclosed group).

Laypersons, HCPs, and physicians accounted for 65%, 28.4%, and 6.6% of the respondents, respectively, after weighting. A total of 66.1% respondents attended conventional CPR training courses, whereas 16.7% respondents learned CPR from nonconventional educational platforms, as shown in Table 1 (weighted disclosed group).

### Attitude and behavior toward BCPR

Among the 1,347 eligible respondents (Table 2), 822 (61%) responded that an EID outbreak had a negative impact on their attitude toward performing BCPR. The proportion of HCPs with a negative attitude (40.7%) was significantly lower than that of laypersons (70.9%) and physicians (74.4%) (p < 0.001). Those with pre-existing CPR training showed a significant difference in attitude change (p < 0.001). The respondents who received CPR training were less likely to have a negative attitude than those without training (74.4%); among the CPR-trained respondents, those who received conventional courses showed a lower rate of negative attitudes (57.1%) than did those who received nonconventional education (67.8%).

**Table 2:** Univariate association between survey predictors and willingness toward BCPR: All eligible respondents.

		Attitude toward BCPR			Behavior(s) toward BCPR					
		Negative changed	Not changed	Total	will not CPR anyway	will CPR w/ instruction	will CPR w/face mask	will CPR w/o MTM	will CPR anyway	Total
		n (%)	n (%)		n (%)	n (%)	n (%)	n (%)	n (%)	
	All eligible respondents	822 (61.0%)	525 (39.0%)	1347	130 (9.7%)	133 (9.9%)	317 (23.5%)	540 (40.1%)	227 (16.9%)	1347
Occupation*										
	Layperson	572 (70.9%)	235 (29.1%)	807	94 (11.6%)	132 (16.4%)	143 (17.7%)	335 (41.5%)	103 (12.8%)	807
	HCP	183 (40.7%)	267 (59.3%)	450	23 (5.1%)	1 (0.2%)	144 (32.0%)	161 (35.8%)	121 (26.9%)	450
	Physician	67 (74.4%)	23 (25.6%)	90	13 (14.4%)	0 (0.0%)	30 (33.3%)	44 (48.9%)	3 (3.3%)	90
Preexisting CPR training*										
	No	131 (74.4%)	45 (25.6%)	176	29 (16.5%)	94 (53.4%)	16 (9.1%)	31 (17.6%)	6 (3.4%)	176
	Conventional course	548 (57.1%)	412 (42.9%)	960	80 (8.3%)	12 (1.3%)	265 (27.6%)	417 (43.4%)	186 (19.4%)	960
	Non-conventional platform	143 (67.8%)	68 (32.2%)	211	21 (10.0%)	27 (12.8%)	36 (17.1%)	92 (43.6%)	35 (16.6%)	211
Attitude toward BCPR**										
	Not changed	0 (0%)	525 (100%)	525	9 (1.7%)	45 (8.6%)	107 (20.4%)	176 (33.5%)	188 (35.8%)	525
	Negative changed	822 (100%)	0 (0%)	822	121 (14.7%)	88 (10.7%)	210 (25.5%)	364 (44.3%)	39 (4.7%)	822

BCPR: bystander cardiopulmonary resuscitation; HCP: healthcare provider; MTM: mouth-to-mouth breathing.

* p < 0.001 for attitude and behavior toward BCPR.

** p < 0.001 for behavior toward BCPR.

Regarding behavioral aspects (Table 2), occupational identity, pre-existing CPR training, and attitude change showed a significant impact on behavior toward BCPR (p < 0.001). If the eligible respondents encountered a stranger who suddenly collapsed and was presumed to have a cardiac arrest, 130 (9.7%) would absolutely refuse to provide BCPR. Those who were more likely to refuse providing BCPR absolutely were physicians (14.4%), respondents not having CPR knowledge (16.5%), and those with a negative attitude toward BCPR (14.7%). On the contrary, 227 (16.9%) eligible respondents reported that they would still perform BCPR despite the epidemic outbreak. Conversely, 133 (9.9%) would perform BCPR if they were instructed; 317 (23.5%), if they were wearing a facemask; and 540 (40.1%), if they were to perform hands-only CPR.

Interestingly, even among the 822 respondents who reported a negative change in attitude toward BCPR, over 85% (n = 701) still tended to perform CPR in certain conditions, including 10.7% (n = 88) if they were instructed, up to 25.5% (n = 210) under facemask protection, over 44% (n = 364) in case there is no need to execute mouth-to-mouth resuscitation, and 4.7% (n = 39) directly performing BCPR anyway. Only 14.7% (n = 121) of the respondents with a negative attitude toward BCPR would absolutely refuse to perform BCPR.

### Weighted subgroup analysis

The associations of attitude and behavior toward BCPR with gender, age, occupation, and pre-existing CPR training for the weighted subgroup analysis are presented in Table 3. The weighted subgroup analysis showed significant differences (p < 0.001) in the attitude and behavior toward BCPR with respect to occupational identity and pre-existing CPR training levels; the results (Table 3) were similar to those in all eligible case analyses (Table 2). In addition, the weighted subgroup analysis showed that men, adolescents aged 10–19 years, and young adults aged 20–29 years tended to have fewer negative attitudes and exhibit more positive behavior toward BCPR.

**Table 3:** Univariate association between survey predictors and willingness toward BCPR: Weighted subgroup.

		Attitude toward BCPR			Behavior(s) toward BCPR					
		Negative changed	Not changed	Total	will not CPR anyway	will CPR w/ instruction	will CPR w/ face mask	will CPR w/o MTM	will CPR anyway	Total
		n (%)	n (%)		n (%)	n (%)	n (%)	n (%)	n (%)	
	Weighted respondents	458 (62.1%)	279 (37.9%)	737	67 (9.1%)	112 (15.2%)	159 (21.6%)	257 (34.9%)	142 (19.3%)	737
Gender*										
	Female	263 (70.9%)	108 (29.1%)	371	31 (8.4%)	88 (23.7%)	74 (19.9%)	132 (35.6%)	46 (12.4%)	371
	Male	195 (53.3%)	170 (46.7%)	366	36 (9.8%)	24 (6.6%)	85 (23.2%)	125 (34.2%)	96 (26.2%)	366
Age group*										
	10–19	33 (41.3%)	47 (58.8%)	80	0 (0%)	3 (3.8%)	24 (30.0%)	17 (21.3%)	36 (45.0%)	80
	20–29	46 (41.4%)	65 (58.6%)	111	4 (3.6%)	4 (3.6%)	27 (24.3%)	42 (37.8%)	34 (30.6%)	111
	30–39	82 (65.1%)	44 (34.9%)	126	5 (4.0%)	6 (4.8%)	36 (28.6%)	59 (46.8%)	20 (15.9%)	126
	40–49	73 (54.9%)	60 (45.1%)	133	9 (6.8%)	20 (15.0%)	27 (20.3%)	56 (42.1%)	21 (15.8%)	133
	50–59	101 (78.3%)	28 (21.7%)	129	19 (14.7%)	27 (20.9%)	26 (20.2%$)	43 (33.3%)	14 (10.9%)	129
	60–69	73 (67.6%)	35 (32.4%)	108	30 (27.8%)	12 (11.1%)	19 (17.6%)	30 (27.8%)	17 (15.7%)	108
	70–79	50 (100%)	0 (0%)	50	0 (0.0%)	40 (80.0%)	0 (0.0%)	10 (20.0%)	0 (0.0%)	50
Occupation*										
	Layperson	347 (72.4%)	132 (27.6%)	479	57 (11.9%)	111 (23.2%)	71 (14.8%)	165 (34.4%)	75 (15.7%)	479
	HCP	73 (34.9%)	136 (65.1%)	209	4 (1.9%)	1 (0.5%)	68 (32.5%)	70 (33.5%)	66 (31.6%)	209
	Physician	38 (77.6%)	11 (22.4%)	49	7 (14.3%)	0 (0.0%)	19 (38.8%)	21 (42.9%)	2 (4.1%)	49
Preexisting CPR training*										
	No	107 (84.3%)	20 (15.7%)	127	15 (11.8%)	84 (66.1%)	10 (7.9%)	15 (11.8%)	3 (2.4%)	127
	Conventional course	279 (57.3%)	208 (42.7%)	487	33 (6.8%)	10 (2.1%)	129 (26.5%)	210 (43.1%)	105 (21.6%)	487
	Non-conventional platform	72 (58.5%)	51 (41.5%)	123	10 (8.1%)	19 (15.4%)	29 (23.6%)	31 (25.2%)	34 (27.6%)	123
Attitude toward BCPR**										
	Not changed	0 (0%)	279 (100%)	279	3 (1.1%)	25 (9.0%)	55 (19.7%)	76 (27.2%)	120 (43.0%)	279
	Negative changed	458 (100%)	0 (0%)	458	65 (14.2%)	87 (19.0%)	104 (22.7%)	180 (39.3%)	22 (4.8%)	458

BCPR: bystander cardiopulmonary resuscitation; HCP: healthcare provider; MTM: mouth-to-mouth breathing.

* p < 0.001 for attitude and behavior toward BCPR.

** p < 0.001 for behavior toward BCPR.

### Pre-existing CPR training levels subgroup analysis

Table 4 showed a subgroup analysis based on the pre-existing CPR training levels of all eligible respondents who are layperson, HCP and physician. Our analysis showed that the only significant difference (p < 0.001) was the behavior toward BCPR of lay respondents with different pre-existing CPR training levels. Among the eligible lay respondents, those who are CPR naïve were more likely to absolutely refuse BCPR (15.5%, n = 27) than those who received conventional CPR courses (10.7%, n = 46) and those who received nonconventional education (10.3%, n = 21).

**Table 4:** Univariate association between pre-existing CPR training levels and willingness toward BCPR.

		Attitude towards BCPR			Behavior(s) toward BCPR					
		Negative changed	Not changed	Total	will not CPR anyway	will CPR w/ instruction	will CPR w/ face mask	will CPR w/o MTM	will CPR anyway	Total
		n (%)	n (%)		n (%)	n (%)	n (%)	n (%)	n (%)	
	**All eligible respondents**	822 (61.0%)	525 (39.0%)	1347	130 (9.7%)	133 (9.9%)	317 (23.5%)	540 (40.1%)	227 (16.9%)	1347
**Layperson**									
	**No**	130 (74.7%)	44 (25.3%)	174	27 (15.5%)	94 (54.0%)	16 (9.2%)	31 (17.8%)	6 (3.4%)	174
	**Conventional course**	301 (70.2%)	128 (29.8%)	429	46 (10.7%)	11 (2.6%)	93 (21.7%)	215 (50.1%)	64 (14.9%)	429
	**Non-conventional platform**	141 (69.1%)	63 (30.9%)	204	21 (10.3%)	27 (13.2%)	34 (16.7%)	89 (43.6%)	33 (16.2%)	204
	p = 0.438			p < 0.001						
**HCP**									
	**No**	1 (100%)	0 (0.0%)	1	1 (100%)	0 (0.0%)	0 (0.0%)	0 (0.0%)	0 (0.0%)	1
	**Conventional course**	180 (40.6%)	263 (59.4%)	443	22 (5.0%)	1 (0.2%)	142 (32.1%)	159 (35.9%)	119 (26.9%)	443
	**Non-conventional platform**	2 (33.3%)	4 (66.7%)	6	0 (0.0%)	0 (0.0%)	2 (33.3%)	2 (33.3%)	2 (33.3%)	6
	p = 0.649			p = 0.251						
**Physician**									
	**No**	0 (0.0%)	1 (100%)	1	1 (100%)	0 (0.0%)	0 (0.0%)	0 (0.0%)	0 (0.0%)	1
	**Conventional course**	67 (76.1%)	21 (23.9%)	88	12 (13.6%)	0 (0.0%)	30 (34.1%)	43 (48.9%)	3 (3.4%)	88
	**Non-conventional platform**	0 (0.0%)	1 (100%)	1	0 (0.0%)	0 (0.0%)	0 (0.0%)	1 (100%)	0 (0.0%)	1
		p = 0.063			p = 0.326					

BCPR: bystander cardiopulmonary resuscitation; HCP: healthcare provider; MTM: mouth-to-mouth breathing.

### Predictors of the attitude and behavior toward BCPR

The predictors of the attitude and behavior toward CPR were analyzed using multivariate logistic regression (Table 5). A positive behavior toward BCPR was defined as the willingness to perform CPR in any of the provided conditions (Table 1: behavior[s] toward BCPR, including those who will perform CPR with instruction, with facemask, and without mouth-to-mouth resuscitation and those who will perform CPR anyway). The younger individuals (OR, 1.26 [95% CI, 1.10–1.45]; p = 0.001), men (OR, 1.77 [95% CI, 1.27–2.46]; p = 0.001), and HCPs (OR, 3.57 [95% CI, 2.46–5.16]; p < 0.001) showed significantly lesser negative change in attitude toward BCPR than did the laypersons. Younger age (OR, 1.64 [95% CI, 1.25–2.14]; p < 0.001) and being an HCP (OR, 2.85 [95% CI, 1.05–7.75]; p = 0.040) were the significant predictors associated with more positive behavior toward performing BCPR compared with being a layperson.

**Table 5:** Multivariate association between survey predictors and willingness toward BCPR.

		Less negative on attitude toward BCPR			More positive behavior toward BCPR		
		OR	95% CI	P	OR	95% CI	P
**Age group** (Younger)		1.26	1.10–1.45	0.001	1.64	1.25–2.14	< 0.001
**Gender** (Male)		1.77	1.27–2.46	0.001	0.91	0.50–1.65	0.743
**Preexisting CPR training** (Yes)		1.16	0.67–1.97	0.580	1.09	0.53–2.26	0.809
**Occupation**							
	Layperson	Reference	n/a	n/a	Reference	n/a	n/a
	HCP	3.57	2.46–5.16	< 0.001	2.85	1.05–7.75	0.040
	Physician	0.68	0.37–1.27	0.225	0.54	0.24–1.20	0.130

BCPR: bystander cardiopulmonary resuscitation; CI: confidence interval; HCP: healthcare provider; OR: odds ratio.

## Discussion

This study noted two major findings. First, it was observed that an outbreak of an EID, such as COVID-19, would have negative impacts on individuals’ attitudes and behaviors toward performing BCPR, especially when the bystander is a physician or had no pre-existing CPR training. Second, we found that among those who already reported a negative change in attitude toward BCPR because of the outbreak, up to 74.5% still expressed positive behaviors toward BCPR (e.g., providing BCPR with facemask protection, performing hands-only BCPR without mouth-to-mouth resuscitation, or directly performing BCPR) once they encountered a cardiac arrest event. These findings could be helpful in exploring the barriers and facilitators of bystander resuscitations during an EID outbreak, which may shape better strategy or guideline modifications for BCPR achievement.

Concerns regarding infectious disease transmission are a well-known barrier to layperson BCPR [29, 30]. In ordinary circumstances, Savastano and Vanni have shown that the fear of infection may be a possible barrier for lay rescuers to perform CPR [31]. In the context of an EID outbreak, Lam et al. reported the adverse effect of a severe acute respiratory syndrome outbreak on bystanders’ willingness to perform CPR [16]. Scquizzato et al. recently reported that laypersons refuse to perform CPR owing to coronavirus fears, thereby deteriorating the community BCPR rate [12]. Similarly, our study results showed that the COVID-19 outbreak has negative impacts on respondents’ attitude and behavior toward BCPR.

Our results indicated that although an individual’s attitude toward BCPR became more negative owing to the COVID-19 outbreak, surprisingly, most of the respondents would be willing to perform life-saving CPR if they encounter a cardiac arrest event. It is commonly assumed that individuals often behave according to their attitudes. However, social psychologists have found that attitude and actual behavior are not always consistent [32]. Our study results suggest that among those who had a negative attitude toward BCPR, only 14.7% of them would absolutely refuse to perform life-saving CPR. Most individuals (over 80% in our study survey) appeared to have attitude–behavior inconsistency such that even if their attitude toward BCPR became negative, they were still willing to perform BCPR in certain circumstances together with safer measures, such as hands-only CPR (44.3%), under facemask protection (25.5%), and under dispatch instructions of guidance (10.7%). Notably, individuals are more willing to perform CPR without mouth-to-mouth resuscitation, regardless of ordinary circumstances [33] or the pandemic context. Our study findings could support the rationality and feasibility of the American Heart Association’s interim COVID-19 guidance for basic life support in adults, especially corresponding to the recommendation of performing at least hands-only CPR after recognition of a cardiac arrest event and covering the nose and mouth of the rescuer and/or patient with a facemask or cloth [14].

It is also noteworthy that in this study, an individual’s attitude and behavior toward performing BCPR were significantly associated with pre-existing CPR training. Our analysis showed that even if the respondents learned CPR from nonconventional educational platforms such as online media, their attitude and behavior toward BCPR were more favorable than those of the respondents without pre-existing CPR training. This finding is consistent with that of previous studies that indicated that CPR training could be a positive predictor of willingness to perform CPR [34].

Furthermore, we also noted relatively poor attitude and behavior toward BCPR among women, elderly population, and physicians. As the willingness to perform CPR is defined as an individual’s perceived likelihood of performing CPR in a future scenario [35], our study findings indicated that these factors would probably be negative predictors of bystander willingness to perform CPR during the pandemic. Several studies have reported that men showed greater willingness toward CPR [36, 37]. It would not be surprising that the elderly population tended to refuse performing BCPR because of physical incapability or knowledge barriers. However, it is of interest that the physician respondents in this study, a group of individuals generally considered with the best knowledge of CPR, had the least willingness to perform BCPR during the outbreak. A similar phenomenon was reported by Huang et al. [36] in a telephone survey aimed to assess the knowledge, attitude, and willingness toward CPR but not specially focused on the occasion of infectious disease outbreaks. We speculated that physicians are more sensitive to uncertain mortality, routes, and risks of transmission of COVID-19, especially at the early stage of the EID outbreak when the evidence on this disease was sparse. Unless proven otherwise, physicians would tend to consider every sudden collapse of a stranger as a suspected COVID-19 infection. Hence, physicians were the least confident or willing to perform BCPR without full personal protective equipment protection (e.g., gown, gloves, N95 respirator, facemask, and goggles or facial shield) that they are used to having in hospital settings [38].

From a governmental policy perspective, measures for public communications, advocacy, and social distancing education through daily or regular press conferences and social media notification operated by governmental epidemic command authority would be common strategies during the epidemic outbreak [21, 25, 26]. These measures could increase public awareness of epidemic risks of COVID-19 and hopefully aid better compliance with personal protective actions. However, they may also increase public fear of disease transmission and have a negative effect on the willingness of laypersons to perform BCPR. Therefore, EMS authorities should simultaneously launch certain countermeasures against this negative effect during the outbreak. Based on our study findings, we recommend the following major countermeasures: (1) continuous monitoring of the BCPR rate, (2) modification and reinforcement of dispatcher-assisted BCPR instructions (e.g., covering the nose and mouth of rescuers and patients with facemasks and emphasizing hands-only CPR), and (3) simultaneous education of the general public to routinely carry an extra facemask for emergency or resuscitations needs. These measures could be prepared for both rescuers and patients to reduce the barriers and fear of BCPR.

### Limitations

This Internet survey study is the first to clearly describe the effect of COVID-19 on the attitude and behavior toward BCPR. However, there were limitations in our study that should be noted. First, our study population comprised active Internet and social media users; therefore, they may not represent the general population. These volunteer respondents from the Internet and social media may have more native attention and experience in first aid and disease outbreak information; hence, they could generally be considered less negatively influenced by BCPR than the general public. Our analysis showed a significantly negative change in the willingness toward BCPR among the study population; therefore, it could be inferred that the perception and reaction of the general public could perhaps be worse. Second, there may be a bias caused by limited Internet coverage and self-selection for data collection from the online survey design. However, there is evidence claiming that online surveys are a promising method for assessing how the general public understands and perceives a fast-moving infectious disease outbreak [39]. To our knowledge, there are 21 million active social media users (88% of the total population) in Taiwan. According to Wang et al. [40], approximately 80% of the Taiwanese population relied on the Internet for COVID-19 information. In addition, in this study, we performed weighting using the raking method in the analysis of the survey data. In the weighted subgroup analysis, we found that the associations of the attitude and behavior toward BCPR with occupation and pre-existing training levels were similar to those of all eligible cases. Third, we were unable to confirm the time from the last CPR training of the respondents and the truthfulness of the respondents’ answers regarding pre-existing CPR training, which could have affected our study results.

The latest number of confirmed COVID-19 cases in Taiwan is very low, which could have also affected our results. However, this study was conducted at the very beginning of the COVID-19 pandemic, that is, within 1 month after the WHO declared a public health emergency of international concern. At this stage, COVID-19 outbreaks had mainly occurred in China, East Asian countries, Iran, Italy, and France. At the time, the cumulative number of confirmed COVID-19 cases in Taiwan exceeded that in the United States. There were four factors that might have stimulated the social pressure and public’s fear of getting infected at this stage: (1) severity of the COVID-19 outbreak in the neighboring countries of Taiwan, especially China; (2) the boarder control and quarantine measures had not been expanded to inbound passengers from countries outside of China, thus increasing the risk of community outbreak; (3) the first COVID-19 mortality case in Taiwan was confirmed at that time; and (4) the public was very sensitive to unknown threats, especially in the early stage of the COVID-19 outbreak, when the evidence on this EID was sparse. For these reasons, we believe that our study results can reflect the public’s reaction and willingness toward BCPR during the COVID-19 outbreak, regardless of the incidence rate in Taiwan.

Finally, our Internet survey was conducted in a single country. There would be international variation in first aid awareness, culture, lifestyle, and societal atmosphere at different stages of the COVID-19 outbreak. Thus, the extrapolation of our study results to other countries should be done with caution. For example, in contrast to our findings, Baldi et al. has observed a significant decrease of the BCPR rate during the first 2 months of the epidemic in Northern Italy [41]. From our perspective, this conflicting information from the more burdened epidemic areas has verified the abovementioned Ajzen’s theory of planned behavior [20]: the willingness to perform CPR is shaped by an individual’s attitude, perceived social pressure, and perception of their capability to perform the behavior. In areas with severe epidemics, the individual’s perceived social pressure such as concerns regarding infectious disease transmission may become more intense, which will have a greater negative impact on BCPR. Given that individual’s attitude toward BCPR is not the only factor that affects “actual” resuscitative actions in the real environment; not to mention that most individuals appeared to have attitude-behavior inconsistency based on our study findings; therefore, we suggest EMS and health authorities should simultaneously launch certain countermeasures against the negative impacts on BCPR during the outbreak.

## Conclusions

Outbreaks of EIDs such as COVID-19 could have negative impacts on public’s attitude and behavior toward providing BCPR. In this study, there were relatively lesser negative impacts on the attitude and behavior among men, younger individuals, and HCPs than among physicians, laypersons, and those without pre-existing CPR training. For those who had a negative attitude toward BCPR during the outbreak, most of them still expressed positive behaviors toward performing BCPR in certain circumstances together with safer measures, such as facemask protection, using hands-only CPR, and under dispatch instructions of guidance. The EMS and health authorities should simultaneously launch certain countermeasures against the negative impacts on BCPR during the outbreak. These findings could be helpful and valuable in exploring the barriers and facilitators of bystander resuscitations during the outbreak to shape better strategy or guideline modifications for BCPR achievement.

## Supporting information

S1 Appendix Questionnaires. (DOCX)
