# Supplementary material for: Attitude and behavior toward bystander cardiopulmonary resuscitation during COVID-19 outbreak
Source: PLoS One. 2021 Jun 23;16(6):e0252841. doi: 10.1371/journal.pone.0252841 (PMC8221461; doi:10.1371/journal.pone.0252841)
Supplement: S1 Appendix — (DOCX) [file pone.0252841.s001.docx]

**S1 Appendix. Questionnaires**

* Required

1. The outbreak of an emerging infectious disease such as COVID-19 has a NEGATIVE impact on my attitude toward bystander CPR. *

⬜ 0. No

⬜ 1. Yes

1. During the outbreak of an infectious disease such as COVID-19, if you encounter a stranger who suddenly collapsed and is presumed to have cardiac arrest and needs CPR immediately (otherwise the person will die). What will your reaction be? *

⬜ 1. Self-protection is a priority. I will not help him/her with any CPR.

⬜ 2. I am willing to help him/her with CPR, but I do not know how to do so and wish
someone could give me the instructions.

⬜ 3. I am willing to help him/her with CPR if I wear a mask.

⬜ 4. I am willing to help him/her with CPR if I need not do mouth-to-mouth breathing.

⬜ 5. Saving life is a priority. I will help him/her with CPR.

⬜ Other:

1. Have you ever taken a cardiopulmonary resuscitation (CPR) training course? *

⬜ 0. No, I have not taken any CPR training course.

⬜ 1. Yes, I have taken a conventional CPR training course.

⬜ 2. Yes, I have taken an nonconventional CPR training course from the Internet.

1. What is your identity? *

⬜ 0. Layperson

⬜ 1. Healthcare providers (including nurses, emergency medical technicians, pharmacists,

dentists, and other medical workers)

⬜ 2. Physician

1. Do you live in Taiwan? *

⬜ 0. No

⬜ 1. Yes

Informed Consent

*This study was reviewed and approved by the Research Ethics Committee of the National Taiwan University Hospital. You are invited to participate in this study voluntarily. Please read this Consent Sheet (Chinese version in* [*https://tinyurl.com/y32ggs4b*](https://tinyurl.com/y32ggs4b)*; English version in*  [*https://tinyurl.com/4b35t76r*](https://tinyurl.com/4b35t76r)*) carefully before participating in this study.*

1. By participating in this study, you are agreeing to provide the most honest answers you can. Any responses you provide will be anonymized, so that neither the research team nor additional respondents will know which is yours. By selecting “I agree,” you are consenting to the condition and Consent Sheet described above. *

⬜ I disagree.

⬜ I agree.

1. I voluntarily agree to disclose my following personal information for the purpose of *research. * (If you choose “No,” then you will skip the questions in the next section and no personal information will be recorded.)*

⬜ No, skip the personal information section.

⬜ Yes.

Personal Information

1. Gender *

⬜ Male

⬜ Female

⬜ Other:

1. Age group *

⬜ < 10 years

⬜ 10–19 years

⬜ 20–29 years

⬜ 30–39 years

⬜ 40–49 years

⬜ 50–59 years

⬜ 60–69 years

⬜ 70–79 years

⬜ > 80 years

1. Email (optional):
2. Phone (optional):
3. Name (optional):

Thank you!

----------------------------------------------------- END ----------------------------------------------------
